# On Amalgam Fillings

**Published:** 1898-09

**Authors:** Thomas Fletcher


ARTICLE IX.

### ON AMALGAM FILLINGS.

THOMAS FLETCHER, F. C. S.

The series of experiments which have now been carried on for a considerable time having brought prominently forward one serious fault existing in a large number of amalgams, to which sufficient attention has not been paid and to which a great number of failures may be attributed, possibly more than may be fairly referred to shrinkage. This fault is a strong tendency to assume a spheroidal form whilst soft, after the filling has been inserted, resulting in a rounding of corners, leaving frequently a considerable space in the most dangerous and unexpected places. As may be imagined, this occurs to the greatest extent when the amalgam is used very soft, and the use of glass tubes of sharp triangular forms shows this fault with many amalgams in a most glaring manner, although they may be practically free from absolute shrinkage in bulk. In the glass tube test suggested by me some twelve months ago, it will be found that many amalgams apparently shrink near the surface in a well-defined ring, which only extends a short distance down. As this, from its purely local character, cannot be entirely attributed to shrinkage a series of experiments was instituted with sharp angular tubes, which at once proved that the greater part of the fault was caused by the alteration of form and the tendency to round off any angles in which the amalgam had been packed.

The power of an amalgam to lie absolutely dead in position when packed is, so far as my experiments go, much more important than the question of moderate shrinkage and one to which especial attention should be paid, any fault in this respect resulting in the immediate penetration of fluids down by any angles to the bottom of the cavity and the lifting of the plug by capillary force. An amalgam of this kind, therefore, can never make a solid plug, and its existence as a protection is short, whatever value it may have as regards to shrinkage.

The defect may be partially remedied in the most imperfect preparations by using them as free from mercury as possible, and by thorough condensation in all the parts of the cavity.—*British Journal of Dental Science.*

